# Potential Role of Traditional Chinese Medicines by Wnt/β-Catenin Pathway Compared With Targeted Small Molecules in Colorectal Cancer Therapy

**DOI:** 10.3389/fphar.2021.690501

**Published:** 2021-07-26

**Authors:** Jinrong Chang, Hoileong Wong Xavier, Dongfeng Chen, Yamei Liu, Hui Li, Zhaoxiang Bian

**Affiliations:** ^1^ School of Basic Medical Sciences, Guangzhou University of Chinese Medicine, Guangzhou, China; ^2^ School of Chinese Medicine, Hong Kong Baptist University, Hong Kong, China

**Keywords:** traditional Chinese medicines, colorectal cancer, Wnt/β-catenin, potential role, small molecules, therapeutic mechanism

## Abstract

Colorectal cancer (CRC) has become a global public health problem because of its high incidence and mortality rate worldwide. The previous clinical treatment for CRC mainly involves conventional surgery, chemotherapy, and radiotherapy. With the development of tumor molecular targeted therapy, small molecule inhibitors present a great advantage in improving the survival of patients with advanced CRC. However, various side effects and drug resistance induced by chemotherapy are still the major obstacles to improve the clinical benefit. Thus, it is crucial to find new and alternative drugs for CRC treatment. Traditional Chinese medicines (TCMs) have been proved to have low toxicity and multi-target characteristics. In the last few decades, an increasing number of studies have demonstrated that TCMs exhibit strong anticancer effects in both experimental and clinical models and may serve as alternative chemotherapy agents for CRC treatment. Notably, Wnt/β-catenin signaling pathway plays a vital role in the initiation and progression of CRC by modulating the stability of β-catenin in the cytoplasm. Targeting Wnt/β-catenin pathway is a novel direction for developing therapies for CRC. In this review, we outlined the anti-tumor effects of small molecular inhibitors on CRC through Wnt/β-catenin pathway. More importantly, we focused on the potential role of TCMs against tumors by targeting Wnt/β-catenin signaling at different stages of CRC, including precancerous lesions, early stage of CRC and advanced CRC. Furthermore, we also discussed perspectives to develop potential new drugs from TCMs *via* Wnt/β-catenin pathway for the treatment of CRC.

## Introduction

Colorectal cancer (CRC) is the third cause of cancer-related death worldwide according to the latest statistics of the International Agency for Research on Cancer (IARC) of the World Health Organization (WHO) (Authors Anonymous, 2021a). It estimated that there are 1.8 million new CRC cases and 880,792 CRC-related deaths in 2018 (Yang et al., 2020). Moreover, the incidence of CRC in some countries is on the rise gradually. Approximately 70% CRC cases are sporadic and develop through the adenoma-carcinoma sequence (De Filippo et al., 2002; Fodde, 2002). Tumorigenesis is usually driven by multiple genetic and molecular alterations in the different stages. The mutations of adenomatous polyposis *coli* (APC) gene, were first discovered as the underlying cause of the hereditary colon cancer syndrome termed familial adenomatous polyposis (FAP); in 1991 (Kinzler et al., 1991; Nishisho et al., 1991). Then some researchers found that APC gene could interact with *ß*-catenin and loss of APC function results in overactive T-cell factor 4 (TCF4)/β-catenin signaling. These findings establish a direct link between Wnt/β-catenin signaling pathway and human CRC. Furthermore, more than 90% of sporadic CRCs has been identified to carry mutations of one or more components of the Wnt/β-catenin signaling pathway including APC based on the genome-scale analysis (Network, 2012). Therefore, the canonical Wnt pathway plays an pivatal role in the development of CRC and may be a significant potential target for CRC treatment.

In clinical practice, standard conventional treatments for CRC are surgery, chemo-therapy and radiotherapy. Currently, with the development of tumor molecular targeted therapy, small molecule inhibitors present a great advantage in improving the survival of patients with advanced CRC. Moreover, long-term application of these therapies can lead to various side effects and toxicities, consisting of nausea, vomiting, mucositis, peripheral neuropathy, and diarrhea (Mcquade et al., 2017). Thus, it is urgent to identify new and more effective drugs for CRC treatment. TCMs have been used for more than 2000 years in China. Owing to the low toxicity and the multi-target capacity (So et al., 2019), TCMs are attracting increasing attention and acceptance for the treatment of CRC as it can alleviate chemotherapy-induced side effects and improve the quality of life of patients with CRC. Previous studies have shown that diverse TCMs exhibit excellent anti-tumor activities in both experimental models and clinical cases. In this review, we focused on ongoing strategies of TCMs used to target aberrant Wnt/β-catenin pathway compared with targeted small molecules as a novel therapeutic intervention in different stages of CRC. Taken together, TCMs will become promising alternative drugs to treat cancer with less toxicity and also be used as an adjunctive treatment together with classic drugs for improving therapeutic outcomes in CRC patients.

### Wnt/β-Catenin Pathway and CRC

**FIGURE 1:**
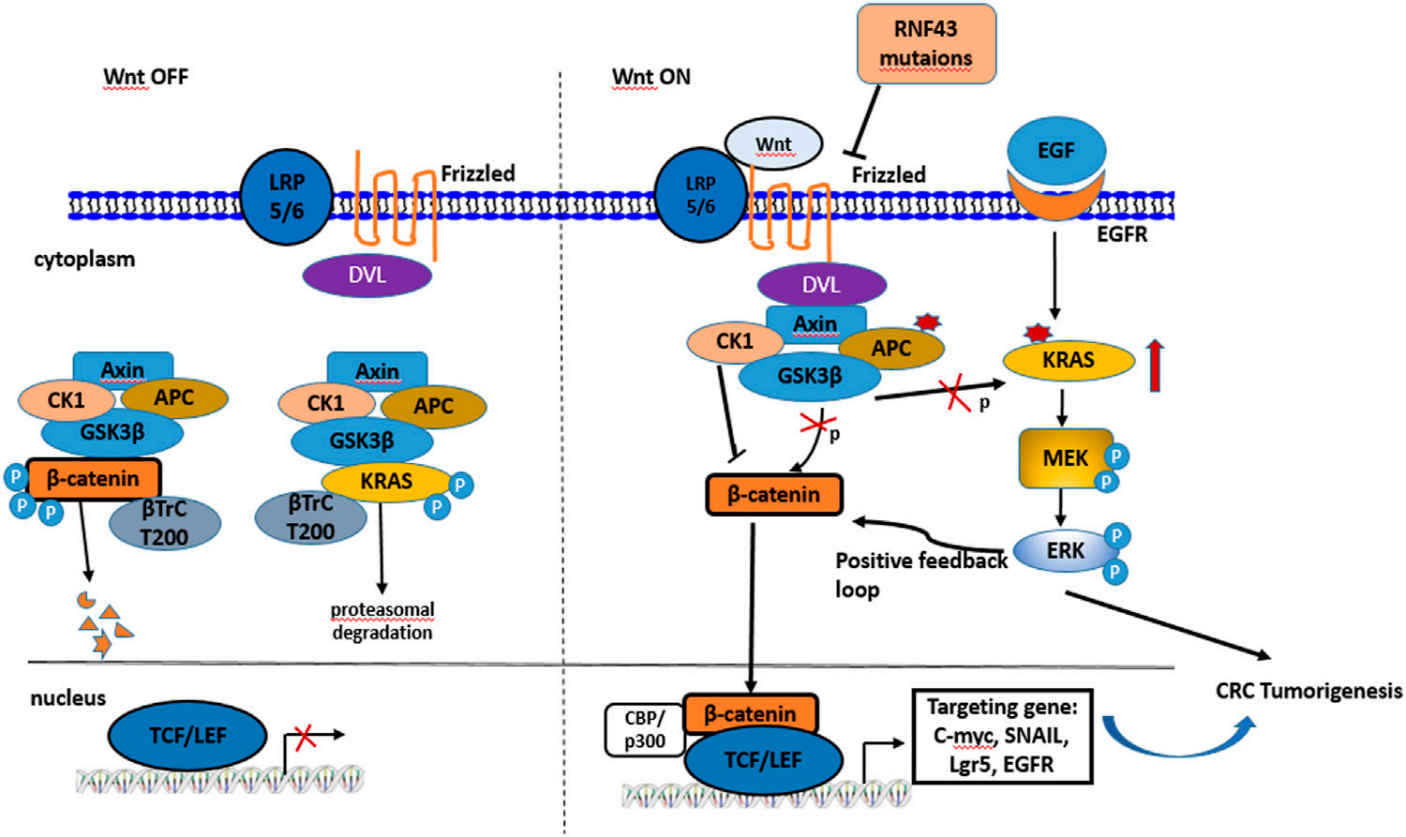
Schematic illustration of the Wnt/β-catenin signaling pathway in CRC. **(A)** Inactive Wnt/β-catenin pathway. In the absence of Wnt ligands, destruction complex phosphylates *ß*-catenin and KRAS for ubiquitination and proteolytic degradation; **(B)** Active *ß*-catenin pathway and crosstalk with KRAS/ERK pathway. In the Wnt stimuli or APC loss, GSK3β becomes inactive status, leading to the high levels of cytoplasmic *ß*-catenin and KRAS. While KRAS mutations have a positive feedback loop with the level of cytoplasmic *ß*-catenin. In addition, RNF43 mutations can relieve the degradation of fizzled protein and activate Wnt/β-catenin pathway.

Wnt signaling pathway is a highly conserved signaling pathway in eukaryotes and commonly divided into canonical (β-catenin dependent) and non-canonical (β-catenin independent) pathways (Polakis, 2012). Originally, many components of the Wnt signaling were identified as key mediators of patterning decisions during embryonic development by genetic screening (Mazzotta et al., 2016). In the last decade, aberrant Wnt/β-catenin pathway activation in carcinogenesis has most prominently been described for CRC. Data from the Cancer Genome Atlas (TCGA) suggests that Wnt/β-catenin pathway is activated in 93% of nonhypermutated CRC and 97% of hypermutated CRC (Li et al., 2012; Sebio et al., 2014; Voorneveld et al., 2015). The status of Wnt/*β*-catenin pathway is mainly related to the stability of *ß*-catenin controlled by the *ß*-catenin destruction complex that is comprised of scaffolding proteins APC, Axin and the kinases casein kinase 1 (CK1) and Glycose synthase kinase 3β (GSK3β). Absence of Wnt ligands stimuli, the cytosolic *ß*-catenin is phosphorylated by GSK3β, ubiquitinated by *ß*-TrCP^200^ and targeted for proteasomal degradation. The ligand Wnt binds to the cell surface receptor Frizzled and low-density lipoprotein receptor-related protein 5/6 (LRP5/6) to form a trimer, which recruits the Dishevelled (Dvl) protein to the plasma membrane, leading to dissociation of the destruction complex followed by cytosolic accumulation of *ß*-catenin. Consequently, the *ß*-catenin translocates to the nucleus where nuclear *ß*-catenin cooperates with TCF/LEF family transcription factors to active target genes such as c-myc, MMP-7, SNAIL and EGFR (Zhan et al., 2017). The activation of Wnt/β-catenin signaling is indispensable for the progression of CRC (Figure 1).

The best-known mutation of APC is the major driver of Wnt pathway in colorectal tumorigenesis which functions as a negative regulator and its importance was further highlighted by several recent studies (Hankey et al., 2018). By using the CRISPR/Cas9 technique to introduce APC mutation into human intestinal organoids, the tumorigenesis of CRC could be modeled *in vivo* (Drost et al., 2015; Matano et al., 2015). Moreover, these studies in human and mouse models indicated that the genotypes of APC mutations are consistent with the distinct levels of canonical Wnt pathway and these alterations are associated with characteristic tumor locations within the large intestine (Buchert et al., 2010; Christie et al., 2013). Besides APC, ring finger protein 43 (RNF43) mutations and R-spondin translocations are noted in over 18 and 9% patients with CRC respectively by preventing removal of Wnt receptor. Both RNF43 and R-spondin fusion are completely opposite to APC mutations (Schatoff et al., 2017). In addition to the well-established function of Wnt/*β*-catenin in CRC, there is accumulating evidence indicating that the KRAS is also an important and frequently mutant gene during colorectal cancinogenesis. Up to 40% of KRAS mutations occur in patients with CRC (Arrington et al., 2012). The discovery of small-molecule RAS inhibitors or a siRNA targeting RAS displayed anti-proliferative activity on xenografts of human CRC cell line SW480 (Song et al., 2020). The mutations of KRAS result in the hyper-activation of RAS-extracellular signal-regulated kinase (ERK) pathway involving transformation of cells and tumorigenesis. Series of studies confirmed the regulation of the RAS-ERK pathway by Wnt/β-catenin signaling and its roles, such as Axin, APC, and GSK3β, and so on (Vincan and Barker, 2008). The crosstalk of RAS and Wnt/β-catenin pathways relies on the phosphorylation of RAS mediated by GSK3β. GSK3β, a key component of the *ß*-catenin destruction complex, is identified as a kinase inducing phosphorylations of *ß*-catenin and RAS at the different sites of the threonine, and subsequently recruits the *ß*-TrCP E3 linker for the proteasomal degradation. Inactivation of GSK3β caused by Wnt stimuli or APC loss further leads to high concentration of cytoplasmic *ß*-catenin and KRAS (Lee et al., 2018a). Therefore, both mutations of APC and KRAS have a positive connection with the Wnt/β-catenin pathway in colorectal tumorigenesis (Figure 1).

Metastasis is a hallmark of advanced cancer and a major challenge to clinic treatment. Epithelial-mesenchymal transition (EMT) is a crucial process by which epithelial cells lose cell polarity and cell-cell adhesion, and closely associate with invasion and metastasis in many types of malignancies including CRC (Spaderna et al., 2006; Vu and Datta, 2017). There is a complicated network involved in the regulation of EMT, containing different signaling pathways. Many investigations indicated that aberrant activation of the canonical Wnt pathway promotes EMT-associated dedifferentiation located at the invasive front of colorectal tumors. Enhanced Wnt/β-catenin signaling in CRC cells induces the action of E-cadherin repressors SNAIL and upregulation of matrix metalloproteinases (MMP) involving CRC invasion and metastasis (Gu et al., 2016). However, inactivating mutations of APC and AXIN2 can up-regulate the canonical Wnt pathway, thereby promoting EMT. Furthermore, *in vitro* and *in vivo* experiments showed that WNT3a overexpression induces SNAIL expression and promotes invasion (Qi et al., 2014).

In addition, increasing evidences suggest that cancer stem cells (CSCs) theory underlies tumor proliferation, differentiation and metastasis. Although there is still no consensus on the concept of cancer stemness, the vital role of the Wnt pathway for the function of normal and cancer stem cells is commonly accepted (Reya and Clevers, 2005). In the intestinal crypt, Wnt/β-catenin pathway exerts a crucial role in the self-renewal of CSCs in CRC (Yan K. S. et al., 2017). R-spondin receptor Lgr5, one putative mark of intestinal stem cells, is a direct target gene of the canonical Wnt signaling cascade and able to promote tumor proliferation after APC is deleted in these cells. The experiments in mouse models showed that the Lgr5^+^ stem cells can increase additionally the population of Lgr5-positive cells and drive adenoma expanding in colon (Barker et al., 2009). CD44v6, as another CSC marker in colorectal cancer, is promoted by Wnt/β-catenin signaling and cytokines secreted from tumor-associated cells (Todaro et al., 2014). Moreover, the tumor environment has an important effect on maintenance of cancer stemness in some studies, such as hepatocyte growth factor, which is secreted by myofibroblasts in tumor micro-environment and can induce stemness features in colorectal cancer cells by improving Wnt activity (Clara et al., 2020). Recently, several studies uncovered potential relations between Wnt pathway and non-coding microRNAs in CSCs. Scientists have discovered miR-142 can inhibit stem cell-like traits by targeting APC gene whose mutations are linked to colon cancer (Isobe et al., 2014). Taken together, these findings indicate that canonical WNT signaling plays a vital role in the maintenance and expansion of CSCs in CRC.

### Small Molecules Targeting Wnt/β-Catenin Pathway for CRC Treatment

Due to the importance of canonical Wnt/β-catenin signaling in human carcinogenic development, small molecule inhibitors targeting Wnt signaling have been developed for the treatment of CRC (Table 1). Activation of Wnt signaling through *ß*-catenin is a critical event in CRC progression. Porcupine (PORCN) is a membrane-bound O-acyltransferase protein which regulates Wnt ligands secretion outside the cell membrane through palmitoylation. In recent years, PORCN has emerged as a molecular target for treating Wnt-driven cancers. ETC-159, WNT974 (LGK974) and Rxc004 has been identified as potent inhibitors of Wnt secretion inhibiting *ß*-catenin activity in preclinical studies. ETC-159 has been proven to be remarkably efficacious in treating CRCs with R-spondin translocation *in vivo* and *in vitro* experiments (Soo and Keller, 2015). During *in vitro* studies in RNF43 mutant and R-spondin fusion CRC cell lines, Rxc004 could potently repress the cell proliferation by arresting cell cycle at G1/S and G2/M phase (Shah et al., 2021). IWP-2 is another inhibitor of PORCN. Experiments on organoid derived from CRC patients unveiled that IWP-2 is sensitive to the cancers with loss of function RNF43 mutations (Masaru, 2017). Pyrvinium, a FDA-approved drug, has been shown to bind to CK1*α* and form a degradation complex with GSK-3, APC, and Axin, resulting in the inhibition of Wnt signaling. Moreover, Pyrvinium suppresses the proliferation of CRC with mutations of APC or *ß*-catenin in HCT116 and SW480 cell lines (Momtazi-Borojeni et al., 2018). ICG-001, a selective inhibitor of Wnt/β-catenin pathway, binds to the CREB-binding protein (CBP) and down-regulates *ß*-catenin/Tcf transcription. As a consequence, ICG-001 selectively induces apoptosis in colon carcinoma cells but not in normal colonic epithelial cells, which is effective in mouse with APC mutations or nude mouse xenograft models of colon cancer. PRI-724, the second generation specific CBP/catenin antagonist for oncology, has been proved to have an acceptable safety profile in early clinical trials and is now under further clinical investigation (Bahrami et al., 2017). Windorphen (WD) is an inhibitor of Wnt/β-catenin signaling by directly targeting p300 to disrupt the association of *ß*-catenin with p300. These findings suggest that WD can selectively kill cancer cells with aberrant activation of Wnt signaling (Hao et al., 2013). Other small molecules, such as NSC668036 and Pen-N3, block the Wnt signaling pathway through binding to the Dishevelled (Dvl) PDZ domain and interrupting the receptor Frizzled (Fz)-Dvl interaction in colon cells (Shan et al., 2005; Zhang et al., 2009).

**TABLE 1:** List of small molecules targeting Wnt/β-catenin pathway for CRC treatment.

Small molecules	Mechanism of action	Preclinical vs. clinical trial (phase) vs. FDA approved	Reference
ETC-159	Porcupine inhibitor	Phase 1	Soo and Keller (2015)
WNT874 (LGK974)	Porcupine inhibitor	Phase 1	Shah et al. (2021)
RXC004	Porcupine inhibitor	Phase 1/2	
IWP-2	Porcupine inhibitor	Preclinical	Masaru (2017)
Pyrvinium	Binding to CK1a	FDA approved	Momtazi-Borojeni et al. (2018)
ICG-001	Binding to CBP	Preclinical	Bahrami et al. (2017)
PRI-724	CBP/β-catenin inhibitor	Phase 1b	
Windorphen	P300/β-catenin inhibitor	Preclinical	Hao et al. (2013)
NSC668036	Binding to Dishevelled	Preclinical	Shan et al. (2005)
Pen-N3	Binding to Dishevelled	Preclinical	Zhang et al. (2009)
XAV939	Tankyrases inhibitor	Preclinical	Huang et al. (2009)
JW55	Tankyrases inhibitor	Preclinical	Waaler et al. (2012)
G007-LK	Tankyrases inhibitor	Preclinical	Lau et al. (2013)
G244-LM	Tankyrases inhibitor	Preclinical	Narwal et al. (2012)
IWR-1	Tankyrases inhibitor	Preclinical	Mashima et al. (2017)
CCT031374	β-catenin inhibitor	Preclinical	Ewan et al. (2010)
CCT036477	β-catenin inhibitor	Preclinical	
CCT070535	β-catenin inhibitor	Preclinical	
iCRT3	β-catenin/Tcf	Preclinical	Gonsalves et al. (2011)
iCRT5	β-catenin/Tcf	Preclinical	
iCRT14	β-catenin/Tcf	Preclinical	
PKF115-584	β-catenin/Tcf	Preclinical	Yan M. et al. (2017)
PKF222-815	β-catenin/Tcf	Preclinical	
CGP049090	β-catenin/Tcf	Preclinical	Tian et al. (2012)
BC21	β-catenin/Tcf	Preclinical	
NC403	β-catenin/Tcf	Preclinical	He et al. (2017)
KYA1797k	GSK3β activator	Preclinical	Lee et al. (2018b)
KY1022	GSK3β activator	Preclinical	Cho et al. (2016)

Some studies indicate that tankyrases (TNKS) are novel targets for Wnt inhibition by regulating stabilization of Axin and hence leading to increased *ß*-catenin degradation. XAV939 and JW55 have been shown to target Wnt/β-catenin pathway through inhibiting the poly-ADP-ribose polymerase (PARP) domains of TNKS in DLD-1 and SW480 cell lines *in vitro* (Huang et al., 2009). JW55 also reduces the growth of tumor in conditional APC mutation mice (Waaler et al., 2012). G007-LK and G244-LM are two other types of small-molecule tankyrase inhibitors (Lau et al., 2013). In particular, G007-LK has greater stability and displays favorable pharmacokinetic properties to inhibit Wnt/β-catenin signaling in APC-mutant CRC xenograft tumors (Tanaka et al., 2017; Katoh, 2018). IWR-1 is another tankyrase inhibitor which interacts with PARP enzyme (Mashima et al., 2017).

β-catenin is a key mediator of Wnt signaling, regulating the stabilization of the destruction complex and consequently intracellular *ß*-catenin levels. Ewan K et al. revealed that three small molecule inhibitors including CCT031374, CCT036477, and CCT070535 can block the Wnt/β-catenin signaling through reducing the level of *ß*-catenin without altering its stability, which is different from drugs involving inhibition of TCF-dependent transcription in SW480 cells (Ewan et al., 2010). Interaction of *ß*-catenin with TCF binding proteins is a crucial step in the activation of target genes in response to the activation of Wnt/β-catenin pathway. A cohort of Wnt antagonists including iCRT3, iCRT5, iCRT14, PKF115-584, PKF222-815, CGP049090, and BC21 have been demonstrated to suppress the Wnt/β-catenin signaling by breaking the association between Tcf4 and *ß*-catenin (Gonsalves et al., 2011; Tian et al., 2012; Yan M. et al., 2017). NC043 is an inhibitor of *ß*-catenin/TCF4, which decreases *ß*-catenin/TCF4 association without affecting the cytosol-nuclear distribution of soluble *ß*-catenin *in vivo* and *in vitro* (He et al., 2017).

In recent years, a small molecular KYA1797K has been identified to suppress the formation of CRCs along with the mutations of APC and KRAS via activating GSK3β and subsequently reducing the level of both *ß*-catenin and Ras as showed both *in vitro* and *in vivo* studies. Moreover, KYA1797K can alleviate the resistant to the EGFR-targeting therapies because of KRAS mutations (Lee et al., 2018b). Whereas, KY1022 destabilizes both *ß*-catenin and Ras by targeting the Wnt/β-catenin signaling in the process of metastasis involving EMT, which is different from the action of KY1797K (Cho et al., 2016). As indicated above, small molecule inhibitors targeting Wnt/β-catenin pathway exhibit promising therapeutic effects on CRC. However, to the best of our knowledge, few of these small molecules has gone into clinical trials. In the future, many scientists will make great efforts to identify more small molecules targeting Wnt/β-catenin and convert them into effective therapies.

### Therapeutic Mechanism of TCMs Against CRC via Wnt/β-Catenin Pathway

It is well documented that uncontrolled cell proliferation is a typical feature in many types tumor development, especially in CRC. The complex balance between proliferation and apoptosis is intimately connected with tissue homeostasis (Diwanji and Bergmann, 2018) and in general, increased cell proliferation along with reduced apoptosis, drives tumor formation. It has been found that many compounds or extracts from TCMs could inhibit colorectal tumorigenesis by targeting different molecules in Wnt/β-catenin pathway. Therefore, we summarized the single-herb and formula of TCM against the different stages of CRC via Wnt/β-catenin pathway (Table 2).

**TABLE 2:** Effects of monomers, extracts, formula of TCMs on CRC by Wnt/β-catenin pathway.

Herbal medicine	Stage	Cell	Animal	Cellular mechanism	Wnt related targets	References
Berberine	Polyps	KM12C	Apc Min/+ mice	Proliferation	β-catenin, APC	Zhang J. et al. (2013)
		KM12SM				
		KM12L4A				
Genistein	Pre-neoplasia	-	SD Rat	Differentiation	Wnt5a, Sfrp1,2,5	Zhang et al. (2020)
EESB	CRC	HT29	BALB/c nude mice	Proliferation Apoptosis	APC, *ß*-catenin	Wei et al. (2017)
Brucine Strychnine	CRC	DLD1, SW480, LoVe	Nude mice	Proliferation Apoptosis	APC, *ß*-catenin, Dkk1	Ren et al. (2019)
Luteolin	CRC	HCT15	BALB/c mice	Proliferation	GSK-3β, *ß*-catenin	Ashokkumar and Sudhandiran (2011)
C. brachycephalum	CRC	SW480	-	Proliferation	GSK-3β, *ß*-catenin	Mervai et al. (2015)
PAG	CRC	HCT116	-	Proliferation, Apoptosis	GSK-3β, *ß*-catenin	Qiu et al. (2017)
					Dvl2	
Wogonin	CRC	SW480	-	Proliferation	GSK-3β, Ctnnb1	Li et al. (2020)
NG	CRC, Migration	HT29, SW620	-	Proliferation Apoptosis, Cell cycle, EMT	GSK-3β, *ß*-catenin	Wen et al. (2019)
IBC	CRC	HCT116, SW480	-	Proliferation Apoptosis	GSK-3β, *ß*-catenin	Li et al. (2019)
4 *ß* HWE	CRC	HCT116, HT29, SW480, LoVo, CCD-CoN-841	BALB/c nude mice	Proliferation, Apoptosis	β-catenin	Ye et al. (2019)
Rg3	CRC	HCT116, SW480	Athymic nude mice	Proliferation	β-catenin	He B.-C. et al. (2011)
Isoquercitrin	CRC	HCT116, DLD-1, SW480	*Xenopus* embryos	Proliferation	β-catenin	Amado et al. (2014)
RTHF	CRC	SW620, HT29	C57Bl/6 mice	Cell cycle, Stemness, EMT	β-catenin	Wu et al. (2018)
TGG	CRC	NIH3T3, HT29	-	Apoptosis	β-catenin	Li et al. (2019)
TET	CRC, Migration	HCT116, SW480	Female athymic nude mice	Proliferation, Apoptosis	β-catenin	He B.-C. et al. (2011)
Curcumin	CRC	SW620, rHCT116	-	Proliferation	β-catenin, Wnt3a	Jiang X. et al. (2019)
				Apoptosis,EMT		
Beta-elemene	CRC	HCT116, HT29	-	Proliferation	β-catenin, Wnt3a	-
				Apoptosis		
Celastrol	CRC	HCT116, SW480	APC Min/+ mice	Proliferation	β-catenin, YAP, LKB1	Wang et al. (2019)
BRB	CRC	HCT116, HT29, LoVo, SW480	c57Bl/6 mice	miRNA	β-catenin, DKK3	Guo et al. (2020)
Quercetin	CRC	SW480, clone 26	-	Proliferation Apoptosis	β-catenin, Tcf4	Shan et al. (2009)
COL	CRC	DLD1, SW480, LoVe	BALB/c nude mice	Proliferation Apoptosis	β-catenin, TCF/LEF	Lei et al. (2019)
Apigenin	CRC	SW480, HCT15	-	Proliferation	β-catenin, TCF/LEF	Xu et al. (2016)
Silibinin	CRC	SW480	Athymic nude mice	Proliferation	β-catenin, Tcf4	Kaur et al. (2010)
Lonchocarpin	CRC	RKO, SW480	*Xenopus laevis*	Proliferation	β-catenin, Tcf4	Predes et al. (2019)
Henryin	CRC	HCT116, SW480, HT29	-	Proliferation	-	Li et al. (2013)
γ-Mangostin	CRC	HCT116, SW480, RKO, LS174T	Nude mice	Proliferation Apoptosis, Stemness	TCF4	Krishnamachary et al. (2019)
Huaier	CRC, Metastasis	T1,T2	-	Stemness	β-catenin, TCF/LEF	Zhang T et al. (2013)
Resveratrol	CRC, Invasion, Metstasis	HCT116, LoVo	-	MMPs	β-catenin	Ji et al. (2013)
IPM711	Invasion, Migration	HT29, HCT116, NCM460	-	Proliferation, EMT	β-catenin, FZD	Ma et al. (2019)
TKP	Invasion, Migration	DLD1, HCT116	-	MMP2, MMP9	GSK-3β	Sun et al. (2020)
Cinnamaldehyde	CRC, Migration	HCT116, SW480	BALB/c nude mice	EMT, Stemness	β-catenin, GSK-3β	Wu et al. (2019)
ZJW	CRC, Invasion, Migration	SW403	-	Proliferation, MMPs	β-catenin, Axin1, Dvl2,3, GSK-3β, Lef1,Tcf4	Pan et al. (2017)
WCA	CRC, Metastasis	HCT116	-	MMPs, EMT	β-catenin	Tao et al. (2019)
HLJDD	CID	HT29	Athymic nude mice	Stemness	Wnt3,Axin2, Fzd5,Pygo2	Chan et al. (2020)
AP	CACC	HT29, HCT116	ICR mice	Proliferation	β-catenin	Li et al. (2020)

Notes: 1. EESB, ethanol extract of Scutellaria barbata D. Don; 2. PAG, pterisolic acid G; 3. NG, Nerigoside; 4. IBC, Isobavachalcone; 5. 4 *ß* HWE, four *ß*-Hydroxywithanolide E; 6. RTHF, Radix Tetrastigma hemsleyani flavone; 7. TGG, 1,4,6-Tri-O-galloyl-β- d -glucopyranose, 8. TET, tetrandrine; 9. BRB, black raspberry; 10. COL, columbamine; 11. IPM711, 4-(1H-imidazo [4,5-f][1,10]-phenanthrolin-2-yl)-2- methoxyphenol; 12. TKP, *Trichosanthes* kirilowii; 13. ZJW, Zuo Jin Wan; 14.WCA,Weichang’an; 15.HLJDD, Huanglian Jiedu Decoction; 16. AP, apple polysaccharide; 17. CID, chemotherapy-induced diarrhea.

## Effect of Active Compounds on Precancerous CRC

The presence of adenoma (polyps), is a precursor and a major risk factor for CRC (Nguyen et al., 2020). Currently, endoscopic removal is the most effective therapeutic regimen for these patients. However, TCMs also have been reported to exhibit important therapeutic effects on colon adenomas. Alkaloid berberine, which is previously used as an anti-inflammatory drug, has proximately been demonstrated to possess anti-tumor activity by reducing Wnt activity and its mechanism of action may involve inhibition of *ß*-catenin translocation to the nucleus by enhancing the expression of APC gene and stabilizing the complex of APC-β-catenin*.* Studies looking at berberine treatment *in vivo* have found that it gave rise to reduced formation of polyps accompanied with a decrease in cyclin D1 and c-myc expression in the intestinal adenoma model. Furthermore, oral administration of berberine has been confirmed to significantly reduce the size of polyps in patients with FAP (Zhang J. et al., 2013). In addition, the discovery of Aberrant crypt foci (ACF) in early colorectal adenomas provided new opportunities to explore the pathogenic mechanism of CRC. Genistein, a soya isoflavone, is capable of decreasing the number of total aberrant crypts in the colon cancer model with azoxymethane (AOM) injection by repressing the expressions of Wnt/β-catenin target genes, including Wnt5a, Sfrp1, Sfrp2, Sfrp5, and c-Myc. These results revealed a novel role for genistein as a suppressor of carcinogen-induced Wnt/β-catenin signaling and the prevention of early colon neoplasia (Zhang et al., 2020).

## Therapeutic Mechanism of Active Compounds Against CRC in Situ

Ninety-three percent of CRC cases has at least one mutation in Wnt/β-catenin pathway genes (Pearlman et al., 2017). The most frequently mutated gene in CRC is APC which may be a promising target for drug development in CRC. The ethanol extract of Scutellaria barbata D. Don (EESB), used for the treatment of various types of cancer clinically (Wei et al., 2017; Zhang et al., 2017; Liu et al., 2018), has been found to prevent the development of human CRC via increasing APC expression with a concomitant decrease in the expression of *ß*-catenin*,* leading to inactivation of the Wnt/β-catenin pathway in a CRC xenografted mouse model and HT-29 cell line. Brucine and strychnine from nux vomic have remarkable effects in improving circulatory system and relieving arthritic and traumatic pains. Recently, Ren H et al. (2019) found both two compounds can suppress the growth significantly by inducing the apoptosis of CRCs in nude mice by enhancing the expression of APC and reducing that of *ß*-catenin. Meanwhile, they can greatly promote DKK1 expression, which is proved to negatively regulate Wnt/β-catenin pathway. On the other hand, some monomers derived from traditional Chinese herbs such as Luteolin, C. brachycephalum, pterisolic acid G (PAG), wogonin, nerigoside (NG) and isobavachalcone (IBC), exhibit anticancer functions by affecting the phosphorylation state of GSK-3β and *ß*-catenin in CRC. However, nerigoside has been found to destroy the balance of proliferation and apoptosis through the ERK/GSK3β/β-catenin signaling pathways, whereas isobavachalcone exerts its anticancer effect via the AKT/GSK-3β/β-catenin pathway in CRC (Ashokkumar and Sudhandiran, 2011; Mervai et al. (2015); Qiu et al., 2017; Li et al., 2019; Tan et al., 2019; Wen et al., 2019).

There are some compounds inhibiting CRC by mediating the core molecule of canonical Wnt pathway. Ye ZN et al. discovered that the anti-tumor effect of four *ß* HWE is to promote the phosphorylation and degradation of *ß*-catenin and the subsequent inhibition of its nuclear translocation in CRC cells (Ye et al., 2019). While, Ginsenoside Rg3 and isoquercitrin were demonstrated to inhibit Wnt/β-catenin pathway by blocking nuclear translocation of the *ß*-catenin protein and hence inhibiting *ß*-catenin/Tcf transcriptional activity (He B. et al., 2011). Moreover, some experiments *in vitro* showed that Radix Tetrastigma hemsleyani flavone (RTHF), 1,4,6-Tri-O-galloyl-β-d-glucopyranose (TGG) as well as tetrandrine (TET) could suppress colorectal tumor growth and downregulate target genes expression (He B.-C. et al., 2011; Wu et al., 2018; Li et al., 2019). Curcumin is another inhibitor of *ß*-catenin in many cancers (Deguchi, 2015). Previous studies illustrated caudal type homeobox-2 (CDX2) is a mediator of the Wnt signaling pathway, and curcumin can reduce cell proliferation and increase apoptosis by restoring CDX2 which inhibited the Wnt/β-catenin signaling pathway (Jiang X. et al., 2019). Besides, curcumin might exert anti-resistant effect of 5-FU on rHCT-116 cells by controlling WNT signal pathway to reverse the EMT progress (Lu et al., 2020). Beta-elemene, however, could elevate sensitivity to 5-FU through down-regulating miR-191 and preventing the Wnt/β-catenin pathway in CRC cells (Guo et al., 2018). Lately, accumulating evidence has strongly suggested Hippo signaling interacted with Wnt/β-catenin pathway. (Jiang Z. et al., 2019). found that celastrol, isolated from Tripterygium wilfordii plant, exerted antitumor effects by accelerating *ß*-catenin degradation via the HSF1–LKB1–AMPKα–YAP pathway in CRC. In addition, miRNA microarray analysis suggested that black raspberry (BRB) anthocyanins can reduce the expression of miR-483–3p accompanied by an increased level of DKK3 expression, which is one negative regulator of Wnt pathway (Guo et al., 2020).

Some studies revealed that quercetin and columbamine (inhibitors of the Wnt/*ß*-catenin pathway) could decrease nuclear lcatenin and downregulate the transcriptional activity of *ß*-catenin/Tcf, leading to inhibition of cell proliferation in SW480 cell lines (Pahlke et al., 2006; Lei et al., 2019). Similar to quercetin and columbamine, apigenin can suppress CRC proliferation by inhibiting *ß*-catenin nuclear entry and thereby prevented the expression of Wnt downstream target genes (Xu et al., 2016). Silibinin and lonchocarpin, also exert anticancer functions through the regulation of *ß*-catenin/Tcf transcriptional activity in animal and cell models (Kaur et al., 2010; Predes et al., 2019). Yet silibinin exhibited selective growth inhibitory effects on SW480 cells (human CRC cells), but not HCT116 cells, by inhibition of Wnt signaling. Henryin, used to control pain for a long time, has been reported to be capable of impairing the association of the *ß*-catenin/TCF4 trans-criptional complex through direct blockade of *ß*-catenin binding to TCF4, but not to affect the cytosol to nuclear distribution of soluble *ß*-catenin (Li et al., 2013). In addition, γ-mangostin, found in Mangosteen fruit, can interact with the transcription factor TCF4 at the *ß*-catenin binding domain, which results in the suppression of the expression of cyclin D1 and c-Myc. Furthermore, γ-mangostin treatment significantly decreased the levels of stem cell markers such as Lgr5, Dclk1 and CD44 in HCT116, LS174T and DLD1 cells, which also confirmed *in vivo* models (Krishnamachary et al., 2019). In the last few decades, the existence of CSCs is central to chemo-resistance and recurrence of many tumors. Some studies identified Huaier aqueous extract can take action against CRC by eradicating CSCs and the Wnt pathway may be considered as a potential target of Huaier for the treatment of CRC (Zhang T. et al., 2013).

## Regulatory Mechanism of Active Compounds Against Metastatic CRC

The development of distant metastases and therefore resistance to therapy, are major clinical problems in the management of the patients with advanced cancer. Recently, medical professionals have focused on TCMs as a way to resolve these issues. Resveratrol, a natural antioxidant from Polygonum cuspidatum, inhibits the invasion and metastasis of human CRC through down-regulation of Metastasis Associated Lung Adenocarcinoma Transcript1 (MALAT1) (Xu et al., 2011; Ji et al., 2013). IPM711, a structurally modified vanillin, was reported to attenuate EMT by increasing the expression of E-cadherin (Ma et al., 2019). Furthermore, a serine protease TKP has a repressive effect on CRC cell invasion and metastasis by targeting MMP2 and MMP9, and is mediated by blockade of both Wnt/β-catenin and Hedgehog/Gli1 signaling (Sun et al., 2020). In addition, cinnamaldehyde has been certified to have potential adjuvant effect on CRC cells in combination with oxaliplatin through blocking the Wnt/β-catenin pathway and enhancing the susceptibility of oxaliplatin in the hypoxic environment (Wu et al., 2019).

## Effect of TCM Formulas on CRC

As well as the monomers and extracts derived from TCMs, an increasing body of evidence suggests that TCM formulas possess anticancer properties, too. Zuo Jin Wan (ZJW) has been used in the treatment of gastrointestinal and liver diseases in China for ages (Chao et al., 2011; Sun et al., 2019), which is composed of Rhizoma Coptidis and Evodia Rutaecarpa at a ratio of 6:1. Berberine and evodiamine are two key elements of ZJW extract and possess anti-tumorigenic activity, respectively (Ayati et al., 2017; Wang et al., 2019). Over the past few decades, many clinical studies had found that some subtypes of 5-HT receptors (5-HTRs) would enhance the proliferation of CRC cells. Recent studies showed that ZJW extracts can exert anti-tumorigenic effects by suppressing the canonical Wnt/β-catenin pathway in animal and cell experiments, similar to that seen with 5-HTR antagonists (Pan et al., 2017). Weichang’ an (WCA) is a traditional Chinese medicinal formula used as an anticancer drug and the experimental data also showed the anti-metastatic function by blunting the activation of Wnt/β-catenin pathway and reducing the expression of MMP9 and the EMT-related protein ZEB1 (Tao et al., 2019). Furthermore, TCM formulations could provide an adjunct for chemotherapy in cancer patients. Huanglian Jiedu Decoction (HLJDD) has been revealed to significantly alleviate the diarrhea induced by chemotherapy in a mouse model. The experiments from the intestinal segments of 5-Fu/CPT-11-treated mice proved pre-treatment with HLJDD could activate the Wnt/β-catenin pathway by inducing the expressions of Wnt signaling components, comprised of Wnt3, Fzd5, Axin2, and Pygo2 (Chan et al., 2020). These data suggest that HLJDD could boost the regeneration of intestinal progenitor cells after chemotherapy, probably by activating Wnt/β-catenin.

In addition, TCMs can also prevent the development of colitis associated colorectal cancer (CACC) through canonical Wnt signaling. It is showed that apple polysaccharide (AP) from apple residues could affect the activation of Wnt/β-catenin signaling pathway *in vivo*, but not *in vitro* experiments (Li et al., 2020). Previous studies showed that AP treatment could effectively decrease the proliferation of *Fusobacterium* in AOM/DSS-induced intestinal tract. Therefore, AP may restrain the activation of Wnt/β-catenin signal pathway in CACC mice through controlling the imbalance of intestinal flora.

### Development of New Drugs in Clinic

CRC is often diagnosed at an advanced stage when tumor cell dissemination has already taken place and chemotherapy was one of the major methods for the treatment of CRC in the past few decades. In clinic, it is obviously clear that fluoropyrimidines, irinotecan, and oxaliplatin have been widely applied to chemotherapeutic regimens for tumors (Gustavsson et al., 2015). The recent introduction of small molecular target agents, such as anti-EGFR (cetuximab, panitumumab) and antiangiogenic molecules (bevacizumab) have led to profound improvements in the life expectancy of patients with advanced CRC (Franke et al., 2019), but with potential lethal adverse drug events and drug resistance. Therefore, it is necessary to develop new and neo-adjuvant therapies in combination with other chemotherapeutics. TCMs and their active compounds with multi-targets was reported to prevent and treat CRC patients as promising candidates, which is distinct from small molecular inhibitors that depend on single target (Yeh et al., 2020). In addition, because of relatively lower toxicity and cheaper price, TCMs can be more accepted by patients with CRC physically and psychologically.

On account of the significance of Wnt/β-catenin pathway in CRC development and metastasis, some native components of TCMs was developed as novel drugs specifically targeting this signaling pathway and are already in clinical trials (Table 3). Resveratrol is a naturally occurring polyphenol with antioxidant, which has been used in many diseases involving cancers. Recently, *in vitro* studies suggest that resveratrol exhibited preventative colon cancer effects and this was associated with Wnt signaling (93). In this clinical trial, patients with colon cancer were randomly provided a treatment with resveratrol, and relevant studies tested its effects directly on colon cancer and normal colonic mucosa. These results showed that resveratrol could inhibit Wnt/β-catenin signaling in the normal colonic mucosa, but not in colon cancer (Nguyen et al., 2009). Thus, resveratrol represented a potential colon cancer preventive strategy in this phase I study. Genistein is also identified to block Wnt/β-catenin signaling and has a cooperative effect with chemotherapeutic agents in lab. According to pre-clinical data, investigators found that combining genistein with standard chemotherapeutic regimens could reduce chemotherapy resistance and improve patient’s response rates (Authors Anonymous, 2021b).

**TABLE 3:** New drugs inhibiting Wnt/β-catenin pathway for treatment CRC in clinic.

New drug	Disease or condition	Combination with	Aim or result	Phase	Recruitment status
Resveratrol	Colon cancer	-	Resveratrol represented a potential colon cancer preventive strategy in this phase I study	Phase I	completed
Genistein	Metastatic CRC	FOLFOX or FOLFOX-Avastin	Combining genistein with the standard of care chemotherapeutic regimens reduced chemotherapy resistance and improved response rates	Phase I and II	completed
Wnt 974 (LGK974)	BRAFV600-mutant Metastatic CRC	LGX818 and Cetuximab	The triple combination of WNT974, LGX818 and cetuximab could result in anti-cancer activity with the inhibition of Wnt and BRAF signals	Phase Ib/II	completed
ABT-888 (veliparib)	CRC that cannot been cured by surgery	Temozolomide	Combining veliparib and temozolomide was well-tolerated at doses up to 200 mg/m^2^/day of temozolomide	Phase II	completed
Foxy5	Metastatic CRC	-	The aim is to set up the recommended drug dose for use in the subsequent clinical phase 2 study and develop Foxy-5 as a first-line drug in anti-metastatic cancer	Phase I	completed
Foxy5	CRC with low Wnt-5a	Surgery to remove the tumour and then giving treatment with FOLFOX about 6 months	In this trial the safety and tolerability of Foxy-5 will be built and early signs of anti-metastatic activity will be evaluated in subjects with resectable colon cancer	Phase II	Recruiting
Niclosamide	FAP	Placebo	Niclosamide has been indicated to have a inhibitory effect on tumorigenesis via inhibition of Wnt pathway with no significant safety issues	Phase II	Recruiting

Besides, small molecular weight Wnt 974, a potential inhibitor of Wnt/β-catenin signaling, has been used to assess its safety and antitumor activity in combination with chemotherapeutic agents in patients with BRAF-mutant metastatic CRC and Wnt pathway mutations (Authors Anonymous, 2021c). Nevertheless, so far, the study results have not been published. ABT-888 (veliparib) has also been used in combination with chemotherapeutic drugs to inhibit the growth of metastatic CRC in phaseⅠandⅡclinical trials (Authors Anonymous, 2021d). But it has not yet been approved by the FDA for use in this cancer. Foxy5, identified by WntResearch, can prevent migration of epithelial cancer cells by mimicking the functions of Wnt**-**5a and thereby play the anti-metastatic role. The safety and tolerability of Foxy-5 were established and early signs of anti-metastatic activity were evaluated in subjects with resectable colon cancer. Further, researchers have already examined the maximum tolerated dose and dose-limiting toxicity of this drug (Authors Anonymous, 2021e). Interestingly, another small molecule niclosamide, an anti-helminthic drug, has been proved to have an obviously suppressive effect on colorectal tumorigenesis by attenuating Wnt/β-catenin signaling lately. In this experiment, investigators devised a double-blind randomized controlled trial to evaluate the effect of niclosamide on patients with FAP. Unfortunately, to date, this project is still in the recruitment stage (Authors Anonymous, 2021f).

## Conclusions and Future Perpectives

CRC has become a global public health problem on account of its high incidence and mortality rate worldwide. The clinical treatments for CRC mainly involve surgery-based chemotherapy. In recent years, with the application of targeting small molecules against cancer, the quality of life for CRC patients has improved. Nevertheless, chemotherapy-induced side effects and drug resistance remain a major issue for clinical practice. Numerous studies have shown that TCMs can be used to exert potential anticancer activity and alleviate the side effects associated with chemotherapy. It is confirmed that various mutations in one or more members of the canonical Wnt signaling pathway take place in the progression of CRC. Therefore, in this review, we aimed to intensively explore molecular mechanisms of TCMs against cancer at the different stages in CRC progress, including precancerous lesions, early stage CRC and CRC invasion and migration based on the inhibition of the Wnt/β-catenin signaling pathway. Cell culture and animal experiments have found that TCMs play anticancer roles by regulating APC/β-catenin, GSK-3β/β-catenin, and *ß*-catenin/TCF4 pathways which represent the main elements of the Wnt/β-catenin pathway involved in the treatment of CRC. Thus, understanding the molecular mechanisms of action of TCMs and how they target Wnt/β-catenin may shed light on future therapies for CRC. However, it needs multi-level and multi-link comprehensive action to anti-tumor because of the complex composition of traditional Chinese medicine. This suggests that we need to investigate the crosstalk between Wnt/β-catenin signal pathway and others. In addition, there remains very few new clinical treatments under development due to lack of strict evaluation system for effectiveness and safety of TCMs. Therefore, it will hopefully pave the way for the CRC clinical treatment and may also relieve the side effects related to chemotherapy if there is a breakthrough in the study of multi-target intervention of TCM in CRC.
